# Sleep in children/adolescents: from self-perception to technical/ economic issues

**DOI:** 10.5935/1984-0063.20210051

**Published:** 2021

**Authors:** Magda Lahorgue Nunes

**Affiliations:** 1 Professor of Neurology, Pontifical Catholic University of Rio Grande do Sul, School of Medicine, Porto Alegre - RS, Brazil.

The present issue of Sleep Science is dedicated to articles related to sleep during childhood and adolescence and it gathered article reporting research from authors living in different regions of the globe.

The definition of “good quality sleep” has been discussed by a multinational group of researchers and its widely known that a good sleep is essential for child development^1^. On the opposite sleep deprivation can compromise the physical and mental health of children and interfere with growth and development^2^. Data regarding sleep disorders in this age period suggests a worldwide prevalence rates ranging from 20 to 40%^3^. In Brazil, a web-based digital survey available to participants from March 2014 to July 2017, showed an overall prevalence of sleep disorders of 25.5%^4^.

Gomes & Martins, from Portugal, analyzed child perception about sleep quality and compared it with parents perception. To do so they have used previously validated questionnaires such as the Pittsburgh Sleep Quality Index (PSQI) that was answered directly by the children, and the Children’s Sleep Habits Questionnaire (CSHQ), answered by the parents. The sample comprised 883 dyads (children and parents). The age range of children varied from 6 to 10 years and they were attending the 1^st^ cycle of basic education either in Lisbon or Leiria, in private and public schools. The results expressed a contradiction between children and parents perception as PSQI revealed good sleep quality and CSHQ poor sleep quality. Authors explored some hypothesis for the reason of this different perception between parents and children^5^. Although interesting, results should be further explored. Does children understood well the questionnaire, does parents reported bad quality of sleep in their children because they, themselves have sleep problems. In two previous studies from our group we have analyzed a similar situation, the influence of maternal aspects on child sleep and the influence of child sleep on parent's sleep. In the first study we have hypothesized that maternal depression in the perinatal period would be associated with poor infant sleep, as results, we have observed that although mothers in the depressed group were more likely to report more night wakings in their children, objective data from actigraphy did not replicate this finding. Suggesting a dysfunctional cognition affecting the mother’s impression of her infant’s sleep^6^. The second study was a web-survey with data collected in the first months of the COVID 19 pandemics in Brazil, besides the findings of an increased prevalence of sleep problems in all age groups studied (children from 0 to 18 years and adults), the fact of having children with sleep disorders was a risk factor for parents worse sleep quality^7^.

The association of sleep duration and physical fitness levels is the topic of a systematic review carried on by Fonseca and collaborators. Based on the previous knowledge that in adult population sleep deprivation has a negative influence on physical activity and sleep restoration improves performance in various sports modalities, authors proposed to investigate this relationship in adolescents and children. After a methodological review of the available literature, 6 articles were eligible for inclusion and those studies reported data on a total of 5,797 participants. Results showed that reduced sleep duration was associated with decreased cardiorespiratory fitness, lower levels of muscular endurance and flexibility just in adolescents and no associations were found for the childhood age range. It is clear from their findings that more studies in children are still necessary to corroborate this association^8^.

Bariani and collaborators, from São Paulo- Brazil, have explored in their article the long-term quality of life related to residual snoring after adenotonsillectomy. Using the OSA-18 questionnaire, that measures quality of life, applied in 25 children divided in two groups , 14 with residual snoring, two or more years after surgery and 11 non-snoring volunteers, they have observed that the snorer group had a worse quality of life compared to the control group. This result brings an important alert to clinicians. Residual snoring should be thoroughly investigated as quality of life may be compromised. Although the sample studied was small, the significant difference in the scores obtained were sufficiently consistent to support the conclusions. Further studies including a snoring non-surgical control group might also add more information to this situation^9^.

Argollo and collaborators presented in their article an interesting case of a 6 year-old girl with a rare respiratory disorder. Catathrenia is characterized by expiratory groans during sleep. In these cases the differential diagnosis with central or obstructive apnea, primary snoring, stridor, sleep-related laryngospasm, sleepiness and parasomnias (somniloquy) is mandatory. The reported patient was misdiagnosed for many years and was considered to have an allergic rhinopathy, she received variable courses of treatment including nasal corticosteroids, oral antihistamines and oral antileukotriene during her lifetime without complete cessation of symptoms. Two red flags were raised with this report, the first how the association of video and audio to the polysomnography helps in the differential diagnosis of more complex situations, and second how a low technical quality polysomnography or a polysomnography that does not comply with the explicit requests in the medical order might delay a sleep diagnosis^10^.

Badaru and collaborators, from Kano city in Nigeria, assessed the prevalence of sleep disorders and its impact on quality of life and exercise participation among children with cerebral palsy. Gross motor function (GMF) , level of spasticity and quality of life were evaluated. Siblings of the case patients served as controls. The prevalence of sleep disorders in cerebral palsy cases was 31.5% and it was influenced by the GMF level. Cases also had significantly more sleep disorders then their siblings. In this interesting study the choice to use siblings as controls was very useful to exclude environmental issues that might disturb sleep. Further it raised the importance of a complete rehabilitation program to help improvement of motor function in order to improve sleep and quality of life in motor disabled children.

Veloso and collaborators, in their article, have investigated the economical burden and technical viability of complete polysomnographies type I (supervised in-laboratory exam) versus Sleep studies type 3. They have investigate 141 children with adenotonsilar hypertrophy and age between 3-11 years. They concluded that although Sleep study type III may have high failure rates and it was a reliable exam for the identification of severe OSA in this population. The cost analysis showed economic feasibility, even with a high failure rate od type III studies and necessity of repetition. They also suggested the necessity of trained persons for installing the device. Despite the high failure rate, the repetition of the failed exam was still economically more feasible than the PSG type I. With the increased interest in sleep disorders and growing demand for their investigation and treatment, investigation solutions that goes beyond the gold standard type I polysomnography must be available for the population, and for this well trained technicians are necessary.

I, together with the editors of Sleep Science, hope you enjoy reading the articles of this issue with many practical and useful information that might help in your clinical practice.
